# Neighborhood socioeconomic status and low‐value breast cancer care

**DOI:** 10.1002/jso.26901

**Published:** 2022-04-22

**Authors:** J. C. Chen, Yaming Li, James L. Fisher, Oindrila Bhattacharyya, Allan Tsung, Samilia Obeng‐Gyasi

**Affiliations:** ^1^ Department of Surgery, Division of Surgical Oncology The Ohio State University Wexner Medical Center and James Cancer Hospital Columbus Ohio USA; ^2^ Department of Biomedical Informatics University of Pittsburg Pittsburg Pennsylvania USA; ^3^ James Cancer Hospital and Solove Research Institute Columbus Ohio USA; ^4^ Department of Economics Indiana University Purdue University Indianapolis Indiana USA

**Keywords:** breast cancer, neighborhood, surgery, value

## Abstract

**Background:**

The objective of this study is to examine the association between neighborhood socioeconomic status (nSES) and receipt of low‐value breast cancer procedures.

**Methods:**

Patients with breast cancer diagnosed between 2010 and 2016 were identified in the Surveillance, Epidemiology, and End Results (SEER) Program. Low value procedures included: (1) axillary lymph node dissection (ALND) for patients with limited nodal disease receiving breast conservation therapy (BCT); (2) contralateral prophylactic mastectomies (CPM); and (3) sentinel lymph node biopsies (SLNB) in patients ≥70 years old with clinically node negative early‐stage hormone‐positive breast cancer. The cohort was divided by nSES. Univariable and multivariable logistic regression analysis compared the groups.

**Results:**

The study included 412 959 patients. Compared to patients in high nSES areas, residing in neighborhoods with low nSES (odd ratio [OR] 2.20, 95% confidence interval [CI] 2.0−2.42) and middle nSES (OR 1.42, 95% CI 1.20−1.56) was associated with a higher probability of undergoing low value ALND. Conversely, patients in low SES neighborhoods were less likely to receive low value SLNB (OR 0.89, 95% CI 0.85−0.94) or CPM than (low nSES OR 0.75, 95% CI 0.73−0.77); middle nSES OR 0.91 (0.89−0.92) those in high SES neighborhoods.

**Conclusion:**

In the SEER Program, low nSES was associated with a lower probability of low value procedures except for ALND utilization.

## 1 INTRODUCTION

Neighborhood socioeconomic composition, social aspects, social capital, disorder, and the built environment influence health behaviors and access to resources which ultimately affect health status and health outcomes.^1^ Among breast cancer patients, neighborhood socioeconomic status (nSES) has been associated with later stages of diagnosis, more aggressive breast cancer subtypes, differences in surgical management, and worse mortality.^2^, ^3^ For instance, women living in neighborhoods with low socioeconomic status are more likely to be diagnosed with advanced stages of disease, triple negative breast cancer (TNBC), and have higher mortality rates than women in areas of high SES.^2^, ^3^ In addition, homogeneous neighborhoods with higher Black resident densities are more likely to exhibit greater communal support with decreased exposure to discrimination, which has been associated with lower risks of developing TNBC.^2^ On the other hand, heterogeneous neighborhoods with lower black resident densities are at higher risk of developing TNBC.^2^ A possible mechanism for the interaction between the environment, tumor biology and clinical outcomes are epigenetic changes such as methylation, histone modification, RNA silencing, and protein folding.^2^, ^4^, ^5^, ^6^ Socially patterned exposures such as lack of social support, social isolation, poor physical conditions resulting in food unavailability, exposure to environmental toxins, and perceived lack of safety have been associated with methylation patterns which contribute to tumor biology.^2^, ^4^, ^5^ Collectively, these studies suggest environmental and social exposures in one's neighborhood have significant implications for breast cancer across the continuum from etiology to survivorship.

In 2012, the American Board of Internal Medicine launched the “Choosing Wisely” campaign in an effort to minimize low‐value care, emphasizing the need to minimize tests, medications, and surgical procedures with no clinically meaningful benefit that cause physical, psychological, and social harm.^7^, ^8^ The American College of Surgeons, Society for Surgical Oncology, and American Society for Breast Surgeons subsequently identified four targets for de‐implementation of low‐value breast cancer care: (1) axillary lymph node dissections (ALND) for patients with limited nodal disease receiving lumpectomies with planned adjuvant radiation; (2) re‐excision of close but negative margins for invasive carcinoma; (3) contralateral prophylactic mastectomies (CPM) for patients with average risk contralateral breast cancer development; and (4) sentinel lymph node biopsies (SLNB) in patients over 70 years old with hormone‐positive breast cancer.^7^ Unfortunately, implementation of the Choosing Wisely Guidelines have been inconsistent with studies showing disparities in implementation secondary to race and facility volume.^7^

To date, no studies have evaluated the implications of nSES on receipt of low value surgical breast care. The purpose of this study is to evaluate the association between nSES and receipt of low value breast surgical procedures. Specifically, we are interested in examining the association between nSES and (1) ALND among patients with clinically T1‐2N0 breast cancer with ≤2 positive sentinel lymph nodes who undergo breast conservation therapy (BCT), (2) CPM among women presenting with unilateral breast cancers, and (3) SLNB among patients age ≥70 with clinically T1N0 hormone receptor positive cancers. We hypothesize that women living in neighborhoods with low nSES are more likely to undergo low value procedures than those living in areas with high nSES.

## 2 MATERIALS AND METHODS

### 2.1 Data source

The Surveillance, Epidemiology, and End Results (SEER) Program is a collection of population‐based central cancer registries capturing facts from 21 geographic areas representing 36.7% of the US population.^9^, ^10^ For this study, the set of 18 SEER Program registries containing census tract attributes was used, in addition to the set of 18 SEER Program registries containing county attributes.^11^, ^12^ Demographic and clinical factors (including surgery and survival/death) of patients diagnosed with breast cancer were obtained from case listing sessions using SEER*Stat software (version 8.3.8).^13^ The numbers of regional lymph nodes examined and found positive were extracted as components of the Collaborative Stage System. The NCI census tract‐level nSES and rurality data, as well as first‐course treatment data reflecting radiation therapy and chemotherapy factors were obtained with permission from the SEER Program. The nSES index in SEER is a time‐dependent composite score. Variables included in the nSES index include education index, median household income, percent below 150% of poverty line, median house value, percent unemployed, median rent and percent working class.^13^, ^14^, ^15^, ^16^ The nSES scores have been categorized into tertiles with the lowest tertile representing a low nSES and the highest tertile representing a high nSES. Census tract‐level rurality data included in SEER are based on the US Department of Agriculture (USDA)'s codes with two categories: Urban area commuting focused (codes 1.0, 1.1, 2.0, 2.1, 3.0, 4.1, 5.1, 7.1, 8.1, and 10.1) and not urban area commuting focused (all other codes).^17^

### 2.2 Low value surgical care

This study focused on three low value procedures: (1) ALND among patients with clinically T1‐2N0 breast cancer with ≤2 positive sentinel lymph nodes who undergo BCT; (2) CPM among women presenting with unilateral breast cancers^18^, ^19^, ^20^; (3) SLNB among patients age ≥70 with clinically T1N0 hormone receptor positive cancers. Performance of ALND among patients meeting ACOSOG Z0011 criteria was first published in 2011 with 10‐year overall survival rates published more recently in 2017.^21^, ^22^ Choosing Wisely advocated for omitting SLNB among women age ≥70 with clinically node negative small hormone positive cancers and avoidance of CPM for unilateral cancer in 2016.^23^, ^24^ Since this study includes breast cancer patients from 2010 to 2017, the study timeframe includes a time period before the guidelines were widely disseminated. However, data supporting omission of CPM and SLNB in the aforementioned populations were available before 2016.

### 2.3 Statistical analysis

The study cohort was stratified into tertiles (low nSES, middle nSES, and high nSES) established by the SEER Program. Sociodemographic, clinical, and treatment variables were recorded; categorical variables were tabulated as frequencies and continuous variables as means with their standard deviation. Pearson's *χ* ^2^ tests and analysis of variance were used, as appropriate, for intergroup bivariable analysis. Odds ratios were calculated to determine the probability of undergoing low value surgical procedures. All *p *values were obtained from a two tailed test. A *p* value of 0.05 was considered statistically significant. The statistical analysis for this study was performed in Stata software Version 16.0 (Stata Corporation).

## 3 RESULTS

### 3.1 Study sociodemographic and clinical characteristics

A total of 412 959 patients were included. Patient sociodemographic characteristics are summarized in Table 1. Compared to those living in areas with high nSES, patients with low nSES notably identified more as Black (26 097/108 148 [24.2%] vs. 6808/16 6295 [4.1%], *p* < 0.001) and reported a single marital status (21 213/108 148 [19.6%] vs. 19 587/166 295 [11.8%], *p* < 0.001). Moreover, those living in areas with low nSES were also more likely to be diagnosed with breast cancer at more advanced stages (distant 8067/108 148 [7.5%] vs. 8188/166 295 [4.9%], *p* < 0.001) and with poorer tumor differentiation (36 399/108 148 [33.7%] vs. 45 634/166 295 [27.4%], *p* < 0.001) than those residing in high SES areas.

**Table 1:** Description of patient sociodemographic and clinical variables based on neighborhood SES

	Total	Low	Middle	High	*p*Value
	*N* = 412 959	*N* = 108 148	*N* = 138 516	*N* = 166 295	
Insurance					<0.001
Uninsured	6639 (1.6%)	2911 (2.7%)	2096 (1.5%)	1632 (1.0%)	
Medicaid	47 313 (11.5%)	23 123 (21.4%)	15 175 (11.0%)	9015 (5.4%)	
Insured	350 326 (84.8%)	79 633 (73.6%)	118 129 (85.3%)	152 564 (91.7%)	
Unknown	8681 (2.1%)	2481 (2.3%)	3116 (2.2%)	3084 (1.9%)	
Age	61.67 ± 13.26	61.73 ± 13.34	62.03 ± 13.27	61.34 ± 13.20	<0.001
Age categorized					<0.001
≤40	23 441 (5.7%)	6535 (6.0%)	7855 (5.7%)	9051 (5.4%)	
41−50	67 168 (16.3%)	16 538 (15.3%)	21 216 (15.3%)	29 414 (17.7%)	
51−60	99 523 (24.1%)	26 446 (24.5%)	32 779 (23.7%)	40 298 (24.2%)	
61−64	45 298 (11.0%)	11 912 (11.0%)	15 398 (11.1%)	17 988 (10.8%)	
≥65	177 529 (43.0%)	46 717 (43.2%)	61 268 (44.2%)	69 544 (41.8%)	
Year of diagnosis					<0.001
2010	55 220 (13.4%)	14 185 (13.1%)	18 467 (13.3%)	22 568 (13.6%)	
2011	57 148 (13.8%)	14 783 (13.7%)	19 223 (13.9%)	23 142 (13.9%)	
2012	58 477 (14.2%)	15 286 (14.1%)	19 660 (14.2%)	23 531 (14.2%)	
2013	59 679 (14.5%)	15 535 (14.4%)	19 990 (14.4%)	24 154 (14.5%)	
2014	59 950 (14.5%)	15 645 (14.5%)	20 322 (14.7%)	23 983 (14.4%)	
2015	61 702 (14.9%)	16 397 (15.2%)	20 590 (14.9%)	24 715 (14.9%)	
2016	60 783 (14.7%)	16 317 (15.1%)	20 264 (14.6%)	24;202 (14.6%)	
Marital status					<0.001
Married/partnered	220 978 (53.5%)	46 609 (43.1%)	72 788 (52.5%)	101 581 (61.1%)	
Separated/divorced	48 599 (11.8%)	15 479 (14.3%)	16 963 (12.2%)	16 157 (9.7%)	
Single	60 528 (14.7%)	21 213 (19.6%)	19 728 (14.2%)	19 587 (11.8%)	
Unmarried/domestic partner	1260 (0.3%)	251 (0.2%)	458 (0.3%)	551 (0.3%)	
Widowed	58 692 (14.2%)	17 964 (16.6%)	20 477 (14.8%)	20 251 (12.2%)	
Unknown	22 902 (5.5%)	6632 (6.1%)	8102 (5.8%)	8168 (4.9%)	
Race/ethnicity					<0.001
Non‐Hispanic White	282 151 (68.7%)	58 255 (54.1%)	97 280 (70.6%)	126 616 (76.6%)	
Non‐Hispanic Black	45 934 (11.2%)	26 097 (24.2%)	13 029 (9.5%)	6808 (4.1%)	
Hispanic	46 248 (11.3%)	18 138 (16.8%)	16 144 (11.7%)	11 966 (7.2%)	
Non‐Hispanic other	36 518 (8.9%)	5246 (4.9%)	11 394 (8.3%)	19 878 (12.0%)	
% < HS education	1367.02 ± 571.23	1621.76 ± 590.82	1322.28 ± 553.81	1238.61 ± 517.19	<0.001
Median family income	7890.14 ± 2014.57	6513.79 ± 1563.16	7638.75 ± 1623.86	8994.63 ± 1946.40	<0.001
Histology					<0.001
Ductal	316 446 (76.6%)	84 113 (77.8%)	107 048 (77.3%)	125 285 (75.3%)	
Lobular	40 608 (9.8%)	9182 (8.5%)	13 134 (9.5%)	18 292 (11.0%)	
Mixed (ductal + lobular)	22 774 (5.5%)	4890 (4.5%)	7205 (5.2%)	10 679 (6.4%)	
Other	33 131 (8.0%)	9963 (9.2%)	11 129 (8.0%)	12 039 (7.2%)	
Stage					<0.001
Localized	269 607 (65.3%)	65,940 (61.0%)	90 061 (65.0%)	113 606 (68.3%)	
Regional	118 802 (28.8%)	34 141 (31.6%)	40 160 (29.0%)	44 501 (26.8%)	
Distant	24 550 (5.9%)	8067 (7.5%)	8295 (6.0%)	8188 (4.9%)	
Grade					<0.001
I: Well differentiated	89 950 (21.8%)	21 010 (19.4%)	30 170 (21.8%)	38 770 (23.3%)	
II: Moderately differentiated	172 889 (41.9%)	42 940 (39.7%)	58 216 (42.0%)	71 733 (43.1%)	
III: Poorly differentiated	123 489 (29.9%)	36 399 (33.7%)	41 456 (29.9%)	45 634 (27.4%)	
IV: Undifferentiated; anaplastic	1381 (0.3%)	398 (0.4%)	471 (0.3%)	512 (0.3%)	
Unknown	25 250 (6.1%)	7401 (6.8%)	8203 (5.9%)	9646 (5.8%)	
RUCA					<0.001
Rural	28 555 (6.9%)	16 807 (15.5%)	10 617 (7.7%)	1131 (0.7%)	
Urban	384 404 (93.1%)	91 341 (84.5%)	127 899 (92.3%)	165 164 (99.3%)	
Surgery type					<0.001
No surgery	21 442 (5.5%)	6695 (6.7%)	6978 (5.4%)	7769 (4.9%)	
Partial mastectomy	212 287 (54.7%)	50 028 (50.0%)	71 163 (54.6%)	91 096 (57.6%)	
Mastectomy	153 697 (39.6%)	43 005 (43.0%)	51 717 (39.7%)	58 975 (37.3%)	
Unknown	983 (0.3%)	353 (0.4%)	363 (0.3%)	267 (0.2%)	
Lymph node surgery					<0.001
None	64 345 (15.7%)	18 980 (17.7%)	21 278 (15.5%)	24 087 (14.6%)	
SLNB	276 635 (67.6%)	66 499 (62.2%)	92 985 (67.7%)	117 151 (71.0%)	
ALND	68 245 (16.7%)	21 516 (20.1%)	23 006 (16.8%)	23 723 (14.4%)	
Radiation					<0.001
No/unknown	216 262 (53.1%)	59 816 (56.2%)	71 905 (52.6%)	84 541 (51.4%)	
Yes with BCS	152 240 (37.4%)	35 093 (33.0%)	51 427 (37.7%)	65 720 (40.0%)	
Yes with mastectomy	38 844 (9.5%)	11 465 (10.8%)	13 255 (9.7%)	14 124 (8.6%)	
Chemotherapy					<0.001
No/unknown	252 374 (61.1%)	62 892 (58.2%)	84 713 (61.2%)	104 769 (63.0%)	
Yes	160 585 (38.9%)	45 256 (41.8%)	53 803 (38.8%)	61 526 (37.0%)	

*Note*: Univariate analysis of cohort stratified by neighborhood SES. Results are listed as *n* (%) with *n *= sample size unless otherwise indicated. Statistical significance is defined as *p* < 0.05.

ALND, axillary lymph node dissection; BCS, breast conserving surgery; HS, high school; RUCA, rural−urban commuting area; SES, socioeconomic status; SLNB, sentinel lymph node biopsy.

### 3.2 Low value surgical care

**Figure 1:**
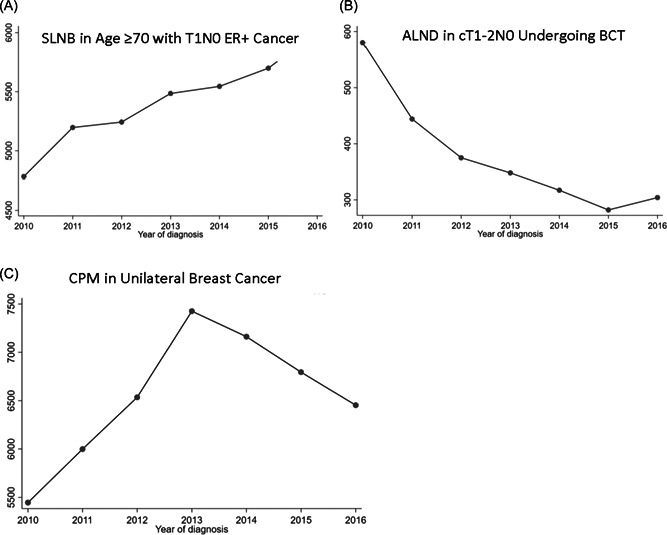
Trends of low value surgery. (A) SLNB n age ≥70 with T1N0ER+ cancer. (B) ALND in cT1‐2N0 undergoing BCT. (C) CPM in unilateral breast cancer.

Over the study period, the number of ALNDs in patients with T1‐2N0 breast cancer and ≤2 positive sentinel lymph nodes decreased. CPMs continued to increase until 2013 when the number of procedures began to decline. However, the number of SLNB in patients ≥70 years old with clinically T1N0 hormone receptor positive cancers continued to increase. Trends of procedures over time are summarized in Figure 1.

When grouped by nSES, as nSES decreased, patients undergoing BCT with T1‐2N0 breast cancer and ≤2 positive sentinel lymph nodes were more likely to undergo ALND (Table 2). Furthermore, there was a trend of increasing SES and higher utilization of two low value procedures: (1) CPM among women presenting with unilateral breast cancer and (2) SLNB among patients age ≥70 with clinically T1N0 hormone receptor positive cancers.

**Table 2:** Evaluation of low value surgery by neighborhood socioeconomic status

	Total	Low	Middle	High	*p*Value
SLNB^a^					<0.001
No	11 635 (23.5%)	2949 (25.3%)	3893 (22.6%)	4793 (23.2%)	
Yes	37 962 (76.5%)	8709 (74.7%)	13 370 (77.4%)	15 883 (76.8%)	
ALND^b^					<0.001
No	111 847 (97.7%)	24 497 (96.4%)	37 668 (97.7%)	49 682 (98.3%)	
Yes	2650 (2.3%)	908 (3.6%)	904 (2.3%)	838 (1.7%)	
CPM^c^					<0.001
No	365 616 (88.9%)	97 533 (90.6%)	122 556 (88.8%)	145 527 (87.8%)	
Yes	45 816 (11.1%)	10 142 (9.4%)	15 462 (11.2%)	20 212 (12.2%)	

*Note*: Frequency of low value surgeries based on neighborhood socioeconomic status. Low value surgeries were defined by the American College of Surgeons, Society for Surgical Oncology, and American Society for Breast Surgeons and include:

^a^ Sentinel lymph node biopsy (SLNB) among patients age ≥70 with clinically T1N0 hormone receptor positive cancers (ER+/PR±/HER2−).

^b^ Axillary lymph node dissection (ALND) among patients with clinically T1‐2N0 breast cancer with ≤2 positive sentinel lymph nodes who undergo breast conservation therapy.

^c^ Contralateral prophylactic mastectomy (CPM) among women presenting with unilateral breast cancers.

On additional analysis, there was an association between decreasing nSES and receipt of ALND in patients with T1‐2N0 breast cancer with ≤2 positive sentinel lymph nodes receiving BCT. Specifically, those living in low SES (OR 2.19, 95% CI [2−2.41], *p* < 0.001) or middle SES neighborhoods (OR 1.42, 95% CI [1.29−1.56], *p* < 0.001) had a higher probability of undergoing ALND than those in high nSES areas (Table 3). Living in a low (OR 0.75, 95% CI [0.73−0.76], *p* < 0.001) or middle (OR 0.90, 95% CI [0.89−0.92], *p* < 0.001) SES neighborhood was associated with a lower probability of undergoing CPM. Patients ≥70 years old with clinically T1N0 hormone receptor positive cancers residing in neighborhoods with low nSES had a 11% reduction in the odds of having SLNB compared to those in neighborhoods with high SES (OR 0.89, 95% CI [0.85−0.94], *p* < 0.001). There was no difference in the receipt of SLNB in patients ≥70 years old with node negative clinically T1N0 hormone receptor positive cancers living in middle and high SES neighborhoods (middle nSES OR 1.03, 95%CI [0.99−1.09], *p* = 0.416; ref high nSES).

**Table 3:** Association between nSES and low value breast surgical procedures

	Odds ratio (95% CI)	*p*Value
SLNB^a^		
Low nSES	0.89 (0.85−0.94)	<0.001
Middle nSES	1.03 (0.99−1.09)	0.146
High nSES	Ref	
ALND^b^		
Low nSES	2.19 (2−2.41)	<0.001
Middle nSES	1.42 (1.29−1.56)	<0.001
High nSES	Ref	
CPM^c^		
Low nSES	0.74 (0.73−0.77)	<0.001
Middle nSES	0.91 (0.89−0.93)	<0.001
High nSES	Ref	

*Note*: Odds of receiving low‐value breast procedures based on neighborhood socioeconomic status (nSES). Low value surgeries were defined by the American College of Surgeons, Society for Surgical Oncology, and American Society for Breast Surgeons and include:

Abbreviations: CI, confidence interval; Ref, reference value.

^a^ Sentinel lymph node biopsy (SLNB) among patients age ≥70 with clinically T1N0 hormone receptor positive cancers (ER+/PR±/HER2−).

^b^ Axillary lymph node dissection (ALND) among patients with clinically T1‐2N0 breast cancer with ≤2 positive sentinel lymph nodes who undergo breast conservation therapy.

^c^ Contralateral prophylactic mastectomy (CPM) among women presenting with unilateral breast cancers.

## 4 DISCUSSION

Although the Choosing Wisely Guidelines were released in an effort to minimize low‐value care, disparities in implementations of these guidelines exist. Study trends suggest that uptake of guidelines on low value surgery are inconsistent. Overall, ALND, and CPM use appear to be decreasing. However, SLNB among older aged adults (age ≥ 70) with clinically node negative small hormone positive cancers continue to rise. When considering the influence of place of residence on receipt of low‐value surgical care, patients living in neighborhoods with low SES are less likely to undergo low value procedures such as SLNB among women aged ≥70 years for clinically node negative small hormone positive cancers or contralateral prophylactic mastectomy for unilateral disease. Conversely, residing in low and middle SES neighborhoods portends more extensive axillary surgeries for small tumors with limited nodal disease within the setting of BCT. Taken together, our results suggest patients' neighborhoods may be a contributor to disparities in low value surgical treatment among breast cancer patients.

Since the release of ACOSOG Z0011 and the Choosing Wisely Guidelines, rates of ALND have decreased from 100% to 17% leading to a 20% reduction in mean overall cost per patient.^25^, ^26^, ^27^ However, the implementation and implications of this uptake on populations that have historically faced barriers in accessing high quality and high value oncologic care, such as marginalized and minoritized individuals, low individual and/or neighborhood SES, is unclear. Emerging studies suggest minority‐serving cancer centers with high‐volume breast cancer cases tend to have similar uptake of clinical trial guidelines compared to academic facilities.^26^, ^28^, ^29^ For instance, Jackson et al. showed no racial differences in receipt of ALND among women meeting ACOSOG Z0011 criteria receiving treatment in high volume centers in the National Cancer Database.^30^ On the other hand, other studies have shown people who face difficulties in accessing oncologic care such as people of color, uninsured patients, individuals with lower levels of education or income are more likely to have ALND over SLNB despite current guidelines.^28^, ^31^, ^32^, ^33^ Similarly, our findings of an association between decreasing nSES and increasing ALND use suggest place of residence may have implications for receipt of some low value breast surgical oncology procedures.

The results on ALND and low nSES are significant as ALNDs have morbidities such as lymphedema, shoulder dysfunction, pain, and numbness, which adversely affect quality of life.^34^, ^35^ Lymphedema following breast cancer impacts up to 35% of breast cancer survivors.^36^ Patients have increased pain, swelling, restricted arm movements, fatigue, and weakness. Symptoms decrease productivity, often leading to job loss and loss of independence within the house.^37^ Patients with lymphedema ultimately face 122% higher monthly costs from medications, specialty lotions, compression garments, and out‐of‐pocket costs which in combination with productivity loss, compromises their ability to manage basic financial responsibilities such as utility bills.^37^, ^38^ The physical and financial burdens of lymphedema often lead to psychological sequelae including concerns about body image and constant reminders of their breast cancer diagnoses and treatments.^39^ Moreover, individuals from minoritized and marginalized groups with lymphedema report even worse outcomes related to mental and physical health and quality of life.^40^ Financial repercussions are often greater in those populations given their disproportionate representation in occupations requiring manual labor with less generous flexibility and benefits.^40^ Combined with higher monthly costs, marginalized and minoritized individuals are placed at an undue burden of economic disadvantage.^41^ Taken together, it can be argued that the financial, emotional, and quality of life cost for patients living in neighborhoods with low nSES undergoing unnecessary ALNDs can be catastrophic.

Although the Choosing Wisely guidelines recommended against CPM in patients with average breast cancer risk, CPM trended upwards until 2013.^42^ CPM rates increased from 4% to 25% from 1991 to 2013, with a sixfold increase from 1998 to 2013, particularly in younger patients who are white, have higher levels of education or income, private insurance, and reconstruction readily available.^42^, ^43^ Rates subsequently decreased from 2013 to 2016, indicating a downturn in CPM utilization in more recent years.^44^ Our results similarly show that CPM increased until 2013 when rates began to decline, coinciding with the publication of the Choosing Wisely guidelines and the American Society of Breast Surgeons CPM guidelines.^7^, ^8^, ^45^ Despite the decline, however, only 42% of patients who have undergone CPM between 2013 and 2018 had justifiable medical reasons such as BRCA^+^or lobular histology.^46^ Younger, more educated patients who received breast reconstruction for the diseased breast tended to undergo CPM with breast reconstruction to allow for symmetry.^47^ Patients with lower income, on the other hand, underwent CPM to avoid needing any additional care given the need to avoid time‐consuming treatments that may jeopardize their financial stability.^47^ Both parties, however, expressed risk of recurrence as one of the primary goals for surgery despite evidence reassuring the low risk of a new contralateral primary breast cancer.^47^, ^48^ Decisions to pursue CPM are also influenced by the stress and anxiety of a new cancer diagnosis and heavily skewed by social media (e.g., Angelina Jolie), family, and friends.^45^ From the surgeon perspective, surgeons report reluctance to decline CPM to patients despite personal preferences due to possible psychologic benefits, patient autonomy, and to avoid any possible repercussions on future referrals or patient trust.^49^

Despite recommendations against SLNB in patients ≥70 years old with clinical T1N0 hormone positive cancer, our results suggest sentinel lymph node biopsy surgery continues to increase over time. Some studies have indicated that as patient age increases, the likelihood of receiving axillary surgery decreases; however, notably more than 60% of patients ≥85 years old still receive axillary surgery.^50^ Interestingly, patients treated at comprehensive community programs had a lower likelihood of receiving SLNB compared to academic programs.^51^ Gunn et al. suggest academic specialists may be more familiar with the nuances of the CALGB‐9343 trial (the basis for omission of SLNB in women ≥70 with small hormone positive cancers), thereby leading to more selective patient criteria to omit SLNB.^51^ Additionally, other specialists, such as radiation or medical oncologist, may advocate for SLNB as omission of nodal tissue leads to less complete pathologic staging which may influence their adjuvant treatment planning.^51^ Nevertheless, recent evidence reports minimal benefit from adjuvant chemoradiation in this population given the higher likelihood of death from comorbidities.^51^ Smith et al. also reports that most surgeons are unaware of these guidelines and are more likely to decide on axillary surgery based on functional rather than chronological age.^49^ Our results, which are consistent with prior studies, indicate that regardless of nSES, the majority of breast cancer patients ≥70 years with small clinically node negative breast cancers are undergoing SLNB. Nonetheless, it is unclear if the slightly lower rates of SLNB in low and middle SES neighborhoods are secondary to increased uptake of guidelines or for other reasons.

While the etiology behind the nSES disparities in implementing the Choosing Wisely guidelines is unclear, patients from minoritized and marginalized groups have historically been found to receive less guideline‐concordant locoregional and systemic treatments.^52^ Some of this has been attributed to increased comorbidities and lack of insurance often seen in communities of color.^52^ However, Black women have also been noted to receive fewer medical oncology consults.^53^ During medical encounters, Black patients also tend to receive less information about their disease, subject to the physician‐patient relationship, thereby hindering self‐advocacy and engagement in their own healthcare.^53^ Similarly, the interplay of multiple social determinants of health (transportation, personal finances, workplace flexibility, stress, etc.) in patients from low SES areas may limit access to institutions that are more likely to follow the most current guidelines.^54^ The results from our study highlight the need for additional research elucidating the pathways between nSES and surgical management. Specifically, qualitative analytical tools such as intersectionality––describes the impact of multiple social identities (e.g., Black race, female gender, and class) on discrimination and disadvantage, provide a much needed contextual framework.^55^ In the setting of surgical disparities based on nSES, frameworks such as intersectionality enables researchers to delve into how social identity that is, race, sexual orientation, socioeconomic status, affects the surgeon−patient relationship, and influences surgical decision making.^56^, ^57^

The strength of this study is the use of a large collection of population‐based central cancer registries. The SEER Program captures information from heterogeneous, diverse geographic areas representing the United States in terms of SES and race and ethnicity.^9^, ^10^ Furthermore, the Yost Index, based on census tract residence, was also used for this study, and because census tracts more closely resemble neighborhoods (as compared to counties), our nSES estimate may allow for a more accurate representation of neighborhood SES.^13^ The limitation of this study is that data points obtained are predefined and are thereby unable to be further clarified when needed. For example, it is not possible for SEER to differentiate with complete accuracy responses of “No” versus “Unknown” for receipt of chemotherapy or radiation; further, there may be biases associated with unmeasured reasons for receiving or not receiving these treatments. Positive pathologic margins are unable to be indicated, yet are often an important prognostic factor in many cancers.^58^

## 5 CONCLUSION

The American Board of Internal Medicine launched the Choosing Wisely Guidelines in 2012 to minimize low‐value care. The present study found that patients with lower nSES are more likely to receive unwarranted and costly axillary surgery. This not only widens the disparity gap, but also significantly increases disparities in postoperative complications and has long‐term implications for quality of life. Notably, some low‐value procedures are less likely in patients with lower nSES, however the underlying driver may be due to inadequate information provided or inferior communication regarding cancer risks and management and not receipt of guideline concordant care. Additional studies are needed to better understand the implications of place of residence on surgical management among cancer patients.

## AUTHOR CONTRIBUTIONS

J. C. Chen, Samilia Obeng‐Gyasi, James L. Fisher, and Yaming Li made contributions to conceptualization, data curation, formal analysis, investigation, writing the original draft, and review and editing subsequent drafts. Allan Tsung and Oindrila Bhattacharyya contributed to the conceptualization, review, and editing of the manuscript. All authors approved the final version of the manuscript.

## CONFLICTS OF INTEREST

The authors declare no conflicts of interest.

## ETHICS STATEMENT

The Ohio State University Office of Responsible Research Practices considered this study exempt from IRB review. There was no consent required as this information was a retrospective review of a database.

## SYNOPSIS

Breast cancer patients living in neighborhoods with low socioeconomic status (SES) are more likely to receive unnecessary ALND. However, other low value procedures such as contralateral prophylactic mastectomies or sentinel lymph node biopsy among women ≥70 years with small hormone positive cancers were more common in higher SES neighborhoods.

## Data Availability

The data that support the findings of this study are available [from National Cancer Institute's Surveillance, Epidemiology, and End Results (SEER) Program. Restrictions apply to the availability of these data, which were used under license for this study. Data are available from the National Cancer Institute's Surveillance, Epidemiology, and End Results (SEER) Program the permission of National Cancer Institute.
